# Assessment of Polypharmacy, Drug Use Patterns, and Associated Factors at the Edna Adan University Hospital, Hargeisa, Somaliland

**DOI:** 10.1155/2022/2858987

**Published:** 2022-08-29

**Authors:** Temesgen Sidamo, Alemu Deboch, Mohamed Abdi, Fikru Debebe, Khalid Dayib, Tamrat Balcha Balla

**Affiliations:** ^1^School of Pharmacy, College of Health Sciences and Medicine, Wolaita Sodo University, Wolaita Sodo, Ethiopia; ^2^College of Natural and Computational Science, Wachemo University, Hossana, Ethiopia; ^3^School of Public Health, Edna Adan University, Hargeisa, Republic of Somaliland, Somalia

## Abstract

Polypharmacy is the use of multiple drugs by a patient at the same time and is common in the elderly. To our knowledge, drug use patterns in Somaliland are rarely studied. The purpose of this study was to evaluate polypharmacy, drug use patterns, and their predictors at the Edna Adan University Hospital in Hargeisa, Somaliland. A retrospective cohort analysis of 1140 medical records and prescriptions over a year from August 28, 2019, to August 27, 2020, was reviewed. The data completeness and consistency were checked and entered with the SPSS version 25.0. The association between total polypharmacy and different variables was analyzed using multivariable binary logistic regression and expressed as an odds ratio (OR) and 95% confidence interval (CI). In addition, the World Health Organization's core drug use and facility indicators were used to assess the drug use patterns. The overall polypharmacy in this study was 71%. Statistically significant association was observed between the polypharmacy and variables such as age (*P* = 0.01; OR = 3.4, 95% CI = 1.9–6.1), chronic illness (*P* = 0.01, OR = 8.6, 95% CI = 5.1–14.7), and comorbidity (*P* = 0.01, OR = 5.2, 95% CI = 2.1–12.9). However, the ward admitted/visited and gender did not have a statistically significant association with polypharmacy. There was overuse of brand drugs (63.9%) and antibiotics (55.2%), while the use of injectables (19.9%) was within the acceptable range. Polypharmacy and overuse of brand drugs and antibiotics were prevalent in the study setting. Essential drugs list, formularies, and treatment and regulatory guidelines are needed in place to ensure appropriate drug use.

## 1. Introduction

Polypharmacy is the use of multiple drugs by a patient at the same time [1]. There is no single definition of polypharmacy as it is still debatable. The World Health Organization (WHO) defines polypharmacy as “the administration of many drugs at the same time or the administration of an excessive number of drugs” [2]. A systematic review of polypharmacy definitions revealed a wide range of variability in polypharmacy definitions and associated terms such as minor and major polypharmacy. It has also been difficult to distinguish between appropriate and inappropriate polypharmacy [3]. Webster's dictionary defines polypharmacy as the practice of administering many different medicines, especially concurrently for the treatment of a single disease [4]. In some literature, taking 2–4 drugs at the same time is classified as minor polypharmacy, whereas taking 5 or more drugs is classified as major polypharmacy [3, 5]. On the contrary, other sources defined the use of 10 or more drugs daily as a major polypharmacy, whereas others still termed it hyperpolypharmacy [6, 7]. Despite the availability of numerous concepts and synonyms in the literature, the phenomenon of polypharmacy remains poorly defined, which has made measuring polypharmacy risks difficult [8].

Polypharmacy occurs when more than one prescription or over-the-counter drug is taken at the same time, and it increases the risk of side effects [9]. It is suggested that while the definition of polypharmacy is numerical, the emphasis should be on evidence-based practice and that the goal must be to reduce inappropriate polypharmacy [10]. Polypharmacy is becoming more prevalent globally, resulting in an increase in adverse drug events (ADRs). According to the International Group for Reducing Inappropriate Medication Use and Polypharmacy (IGRIMUP), an urgent integrated effort to reduce inappropriate medication use and polypharmacy should be a top global priority [11].

Comorbid conditions make polypharmacy more common in elderly patients. Combinations of two to three different drugs, for example, are prescribed in the case of diseases such as heart failure and high blood pressure [12, 13]. Drugs are taken for a long time for chronic diseases, so ADRs are common. This, in turn, necessitates treatment with other drugs, which may be the cause of polypharmacy [13]. When patients have coexisting diseases, the number of drugs per prescription increases, as does the risk of ADRs. Drug interactions (DDIs) are one of the potential causes of ADRs in polypharmacy [14].

DDI has a two-way impact on the final outcome. On the one hand, it may raise the serum levels of the drugs involved, resulting in toxicity or ADR. In contrast, DDI can result in subtherapeutic serum drug levels and, as a result, treatment failure. Taking more than five medications, therefore, increases the likelihood of such interactions [15]. It is very difficult to determine which drug is responsible for ADR, but the ability to predict DDI is important to ensure the safety of patients receiving multidrug treatment. Various machine learning-based computational approaches have been developed nowadays to predict DDI [16].

Rational use of drugs is currently a global issue for the optimal benefit of patients [17]. It requires safe, effective, and affordable access to medicines for all patients who need them for effective management of the disease. Irrational drug use is a big problem all over the world. More than half of all drugs are improperly prescribed, dispensed, or sold. Over half of all patients take drugs inappropriately [18]. The situation is exacerbated in developing countries, where less than 40% of patients in the public sector and less than 30% in the private sector are treated according to clinical guidelines [18, 19].

According to WHO, irrational drug use includes the use of too many drugs per patient; inappropriate use of antimicrobials, often in inadequate dosage for nonbacterial infections; overuse of injections while oral formulations would be more appropriate; failure to prescribe in accordance with clinical guidelines; inappropriate self-medication, often of prescription-only drugs; and nonadherence to dosing regimens [19]. Antimicrobial resistance (AMR) is a serious global challenge that can hamper the progress made by modern medicine in the fight against infectious diseases [20]. Excessive and indiscriminate use of antibiotics, which are surrogate markers for AMR, is common in low-income environments [21]. AMR is ubiquitous and can affect people of all ages in any country in the world. Evaluation and appropriate action plans to mitigate AMR and ensure safe and proper use of drugs in all healthcare systems are a global challenge [22].

WHO's core drug use indicators can be used to assess the rationality of drug use in a medical setting. The number of drugs prescribed per encounter, the percentage of drugs prescribed by brand name, the percentage of antibiotics and injectable drugs prescribed per encounter, and the percentage of drugs prescribed from the list of essential medicines are WHO's core drug use indicators [23, 24].

The Republic of Somaliland is a self-declared state, internationally considered as an autonomous region of Somalia [25]. To the best of our knowledge, drug use patterns in Somaliland are rarely studied. There was no strict regulatory system for drug use in this area, probably because it was the Horn of Africa devastated by the war. In addition, dosing records and prescriptions are poorly organized in most of the medical facilities in this setting [25, 26]. However, the Edna Adan University Teaching Hospital has relatively well-documented medical records. As far as we know, there is little previously examined evidence of polypharmacy and patterns of drug use in Somaliland. Therefore, this study aimed to assess factors and drug use patterns associated with the degree of polypharmacy based on medical records and prescription papers from Edna Adan University Hospital.

## 2. Materials and Methods

### 2.1. Study Setting and Design

Edna Adan University Hospital is a charitable, nonprofit hospital in Hargeisa, Somaliland. Edna Adan Ismail, former Secretary of State and former Somali first lady, founded the hospital. Edna Adan Ismail donated a United Nations (UN) pension and other personal assets to address serious health problems that threaten the lives of women and children in the Horn of Africa [27, 28]. Patient files and prescriptions stored at the Edna Adan University Hospital Pharmacy over a one-year period (between August 28, 2019, and August 27, 2020) were evaluated in this study.

The study used a retrospective cohort design. We included all patient medical records and prescription papers, as well as required patient and prescription information written in easy-to-read handwriting. Patient cards that were medically evaluated during data collection, prescriptions without patient records, or patient records that were missing or with unreadable handwriting were excluded. A total of 1140 medical records and prescription papers from both the outpatient (OP) and inpatient (IP) wards were included by a purposive sampling method. Information about the total number of drugs per prescription, antibiotics prescribed per encounter, brand name usage per prescription, and injectable drugs per prescription were collected.

### 2.2. Data Collection

Demographic characteristics (age and sex), admitted/visited ward, disease condition and diagnosis, presence of comorbidity, and the WHO drug use indicators were collected by nurses and pharmacy technicians. The data collectors were oriented and informed about the study design and its purpose prior to the data collection. A predesigned checklist and the World Health Organization (WHO) drug use assessment formats (Supplemental files 1–3) were used to extract relevant patient data for our study. The data abstraction format was pilot-tested prior to the actual data acquisition in 5% of the total study samples. Initially, the medical records were not categorized as outpatient and inpatient wards because they were all stored in one place. Therefore, we sorted the files according to the wards to which the patients were admitted or visited before the actual data collection. The initial data abstraction format also considered only the number of drugs per prescription less than or equal to 5, while the number of drugs prescribed in the medical records showed up to 10 drugs per prescription in some of the patient records. Thus, the final data abstraction format was changed to incorporate these aspects (Supplement file 1). Key WHO drug use indicators such as the number of drugs per prescription, the presence of at least one antibiotic, injectable drugs, and brand name drugs were also collected as per the WHO format (Supplemental file 2). Each of these WHO drug use indicators was evaluated against recommended reference values (Supplemental file 3) [24]. In addition, we evaluated the most frequently diagnosed illnesses and frequently prescribed drugs in the hospital. The accessibility of WHO facility indicators such as the essential drug list (EDL), standard treatment guidelines (STG), and national drug formularies was also evaluated.

### 2.3. Operational Definitions

In this study, the following terms were operationalized. Polypharmacy (minor plus major) was defined as the prescription of at least two drugs per encounter. Minor polypharmacy refers to prescriptions that contain two to four drugs per encounter, whereas major polypharmacy contains five or more drugs per encounter. Comorbidity was defined as the coexistence of two or more illnesses in the medical records of the patients reviewed. Acute disease in this study refers to a health condition that is short-lived (e.g., acute infection and some temporary symptoms such as diarrhea, cough, and fever) for which the patients visited or admitted to the hospital otherwise the patients were previously healthy. Chronic disease refers to a condition that requires long-term treatment (e.g., cardiovascular diseases, hypertension, diabetes mellitus, and asthma), and the patients visited or admitted may be new or with a previous treatment history. The inpatient (IP) ward in this study is where the patients are admitted and under the direct care of the healthcare professionals in the hospital for a specific period, whereas outpatient (OP) refers that the patients who visited the hospital for their health condition and leave the hospital after receiving treatment and medications for their diagnosis.

### 2.4. Statistical Analysis

After checking for completeness and consistency, the data were transferred to the Statistical Package for Social Sciences (IBM SPSS Statistics for Windows, Version 25.0, Armonk, NY: IBM Corp.). Key WHO drug use indicators were descriptively analyzed for proper use and scored against the WHO recommendations. The association between total polypharmacy (minor plus major polypharmacy) and independent patient-related variables was analyzed using multivariable binary logistic regression and expressed as an odds ratio (OR) and 95% confidence interval (CI). The independent variables evaluated for their association with polypharmacy included patient conditions (acute or chronic disease), comorbidity, age, gender, and the ward to which patients were admitted (inpatient) or visited (outpatient). The level of statistical significance was set at *p* < 0.05. The data were summarized using a bar chart, tables, and a pie chart.

### 2.5. Ethics Statement

Access to ward medical records and pharmacy prescriptions is ethically endorsed by the School of Pharmacy at Edna University. The name and patient ID have been removed to ensure the confidentiality and anonymity of the patient's medical records.

## 3. Results

### 3.1. Demographic Characteristics of Patients

**Figure 1:**
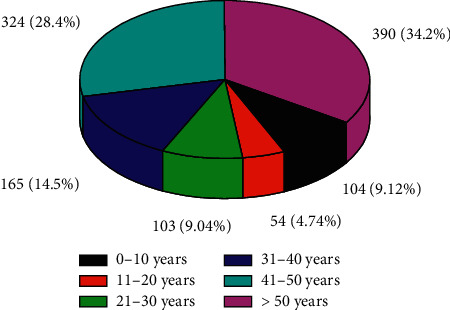
Age distribution of the patients (*N* = 1140) whose medications and prescriptions were involved in the study at the Edna Adan University Hospital, between August 28, 2019, and August 27, 2020.

The age distribution of the patients is shown in Figure 1.

Prescriptions were classified according to the patients' disease conditions. Disease conditions in association with polypharmacy include acute and chronic cases. The proportion of females (822, or 72.1%) was higher than that of males (318, or 27.9%). At the two wards, 379 inpatients and 761 outpatients were admitted and treated. The mean age ± standard deviation (SD) of the patients included in this study was 41.2 ± 0.50. The median age (interquartile) of the patients was 40.0 (32.3–54.0).

### 3.2. Level of Polypharmacy and Associated Factors

**Figure 2:**
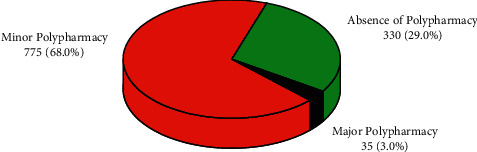
Levels of polypharmacy based on the prescribed medications (*N* = 1140) at Edna Adan university, between August 28, 2019, and August 27, 2020.

As it is depicted in Figure 2, the overall level of polypharmacy was 71%. Of these, a total of 775 (68%) prescriptions containing 2 to 4 drugs per prescription constitute minor polypharmacy, whereas only 35 (3%) prescriptions containing 5 or more drugs constitute major polypharmacy.

Table 1 shows the association between polypharmacy and the ward admitted (inpatient versus outpatient), disease condition (acute versus chronic), age (<40 versus ≥40), comorbidity (yes versus no), and gender (male versus female). There was a statistically significant (*P* < 0.05) association between polypharmacy and disease conditions, comorbidity, and age. However, the ward in which the patients were admitted or visited and the patient's gender did not show a statistically significant association with polypharmacy.

### 3.3. WHO's Core Drug Use Indicators

Drug use patterns were assessed using the WHO core drug use indicators [24]. The description of the total number of drugs per prescription is shown in Table 2. Prescriptions containing 1, 2, 3, 4, 5, 6, and 10 drugs per prescription were 29.0, 37.8, 22.7, 7.2, 1.9, 0.6, and 0.6, respectively.

The average number of drugs per prescription ± standard deviation (SD) and median (interquartile range) was 2.2 ± 0.04 and 2.0 (1.0–3.0), respectively. The percentages of prescriptions for injectable drugs, brand name drugs, and antibiotics are shown in Table 3. The total number of drugs prescribed by brand name was 63.8 percent, with antibiotics accounting for 55.2 percent of the total. The total number of injectable agent prescriptions was 19.9 percent.

### 3.4. The Most Common Diagnosis and Prescribed Drugs

Table 4 lists the most common diagnoses and drugs prescribed at Edna Adan University Hospital. Antibiotics and infectious diseases were among the most common diagnoses for which drugs were prescribed. Antibiotics account for 55.2 percent of all prescriptions. The type of infections diagnosed were urinary tract infections (UTIs), and unspecified infections, wound infections, vaginal infections, skin infections, oral candidiasis, and the proportion of antibiotics prescribed were 6.8%, 24.9%, 9.9%, 7.1%, 4.1%, and 2.4%, respectively. Antibiotics such as amoxicillin, Augmentin® (amoxicillin and clavulanic acid), and ceftriaxone were empirically prescribed for unspecified infections based on clinical symptoms reported by patients, such as sore throat, cough, and unknown fever.

As shown in Table 4, antihypertensive drugs were the second most commonly prescribed drugs, accounting for 10.5% of all cases. These include nifedipine (2.2%), losartan (2.2%), enalapril (2.4%), propranolol (2.1%), and furosemide (1.6%). Drugs for gastrointestinal disorders (GI upset) were the third most commonly prescribed (9.7%), followed by antianemics (8%), antidiabetics (7.5%), hormonal drugs (6%), painkillers (2.1%), and anti-asthmatic drugs (0.9%).

### 3.5. The WHO Facility Indicators

WHO facility indicators such as the essential drug list (EDL), national drug formulary, and standard treatment guidelines were not available at Edna Adan University Hospital. In addition, at the time of this study, there were no locally established, adopted, or adapted regulatory guidelines.

## 4. Discussion

This study showed overall polypharmacy is prevalent though the majority were minor polypharmacy in the study setting. Possible reasons for polypharmacy are lack of understanding and education, differences in medical care, lack of strict regulatory systems, empirical and benevolent treatment, differences in socioeconomic status, and characteristics of population morbidity and mortality [29]. According to the renowned alchemist Paracelsus, “everything is poisonous and nothing is nonpoisonous.” “The dose makes it poisonous or curative.” [29]. In general, more than one drug taken at a time, whether prescription or over-the-counter, poses a risk of drug interactions and side effects. Similarly, polypharmacy may be a reason for the wastage of essential drugs, wrong drug use, overuse, and underuse. Polypharmacy could be a cause of drug-related death in patients. Such deaths could be related to drug interactions that may lead to unanticipated adverse or toxic effects. However, many of those deaths continue to be undetected or unnoticed [29, 30].

In our study, major polypharmacy (3%) was lower, whereas total polypharmacy (71%) was higher than in similar studies in other settings. Polypharmacy (the use of five or more drugs) was found to be 24.1% in an older patient polypharmacy study in western Ethiopia [31]. A recent meta-analysis of the elderly Ethiopian population found 33% polypharmacy [32]. A polypharmacy study in South-West Nigeria found 23.8% total polypharmacy, which is higher than the major polypharmacy but lower than the minor polypharmacy in our study [33]. A similar study from Saudi Arabia found an overall polypharmacy rate of about 51.5%, which is significantly higher than our study finding for a major polypharmacy [34].

Polypharmacy was found to be strongly related to patient age, chronic disease, and comorbidity in this study. Studies conducted elsewhere [35, 36] support the association between age and polypharmacy. Similarly, the relationship between multiple and chronic diseases has been extensively discussed [13, 37]. However, as in other studies, gender had no effect on polypharmacy in our study [34].

The use of branded drugs is slightly different from what WHO recommends [24]. As it is indicated in Table 3, prescriptions that did not contain branded drugs were 36.2%. This is significantly lower than the survey report (90.61%) of selected public hospitals in East Ethiopia. Some of the brand name drug prescribing issues are unnecessary costs, difficulty remembering the name of the drug, accessibility issues, and bioequivalence mismatches. The solution to these problems is to promote prescription writing by generic names that avoid the risks associated with brand name prescribing [30].

The use of antibiotics in this study was unacceptably high (55.2%) compared with the WHO standard (20–26.8%). In fact, the overall assessment of the patients admitted to the hospital showed that infectious disease was the most prevalent case (Table 4). This could be a probable reason for the observed overuse of antibiotics. Interestingly, the highest proportion of antibiotics prescribed (24.9%) was for nonspecific infections. Also, of 10 most commonly used drugs, the first 6 were antibiotics. The reason why antibiotics use was high at the Edna Adan University was unclear and beyond the scope of this study. Studies in selected public hospitals in eastern Ethiopia also revealed that antibiotic use was more widespread [31]. This suggests that the abuse and indiscriminate use of antibiotics is a common trend and the regulatory system is loose in the East Africa. The misuse of antibiotics and the consequent development of resistance to antibiotics is a global problem. In general, self-medication due to a loose regulation and lack of education is one of the reasons for antibiotic abuse. Antibiotic overuse is a major factor in the incidence and prevalence of antibiotic resistance [38–41]. Reports disclosing resistance of serious antibiotic medicines such as vancomycin, which serve as reserves, are emerging [42]. Therefore, it is imperative to look seriously into the rational use of antibiotics.

In fact, and fortunately, the use of injectable medicines in this study was within the WHO recommended range (13.4–24.1%). This is a good habit and a tendency that must be maintained to maximize patient safety. The risks of accidental infections, patient injuries, and deaths associated with injectable drug mistakes are well known [43–45]. Less frequent use of injectable drugs keeps these risks minimal. Conversely, if the injectable form consists of multiple-dose vials, there is an increased risk of inaccurate dosing and contamination that can lead to accidental infections [46]. According to a 2007 American Nurses Association (ANA) study of injectable medication errors, 99% of nurses are at serious risk to their patients when they occur, with nearly half (48%) of the errors. We believe it is most likely to occur during treatment preparation and administration of intravenous (IV) drug [47, 48].

Also, there were no key drugs identified at the hospital level. The absence of any of these facility indicators was the gap observed in this study. There were no key drugs established at the hospital level.

The practical implication of this study would be an appropriate use of drugs according to the WHO recommendations with regard to the number of medicines per encounter and antibiotics, brand medicines, and injections. To this end, the WHO facility indicators such as essential drug lists, established key drugs, standard treatment guidelines, national formularies, and regulatory guidelines are needed to avoid unjustified polypharmacy and to practice rational use of medicines. Further research in the other hospitals in the country remains important for effective intervention and understanding of the root causes. Moreover, the association between polypharmacy and mortality is recommended to be investigated with improved research methods to enhance the reliability.

### 4.1. Limitation of the Study

The association (cause-effect relationship) between polypharmacy and the potential mortality and morbidity was not investigated, and it could be taken as a limitation. In addition, since the study was based on a single private hospital in Somaliland, which is the biggest one, there might be a slight patient selection bias because a referral is a precondition for a patient to visit the hospital. Hence, the findings might be questionably generalizable to the health facilities and populations in Somaliland. Moreover, as the study was planned and commenced before the emergence of the COVID-19 pandemic, the potential association of COVID-19 with the findings was not considered.

## 5. Conclusions

As per the findings, polypharmacy is prevalent in Edna Adan University Hospital. Age, chronic disease, and comorbidities are highly associated with polypharmacy. Moreover, there is significant overuse of brand formulations and antibiotics. The use of injectable medicines remains within the recommended range of the World Health Organization. It has been revealed that chronic disease conditions and the presence of comorbidity are positively associated with polypharmacy as is age of 40 years and above. However, polypharmacy did not show any significant differences across gender and ward variations.

## Figures and Tables

**Table 1:** Association between overall polypharmacy (*N* = 810) and predictors at Edna Adan University Hospital from August 28, 2019, and August 27, 2020.

Variables		Frequency/total encounters (%)	Polypharmacy/total (%)	*p* value, OR (95% CI)
Disease condition	Acute	318/1140 (27.9)	21/318 (6.6)	*p*=0.01^*∗*^, 8.6 (5.1–14.7)
	Chronic	822/1140 (72.1)	789/822 (96.0)	

Ward	Inpatient	379/1140 (33.2)	276/379 (72.8)	*p*=0.34, 0.8 (0.5–1.5)
	Outpatient	761/1140 (63.8)	534/761 (70.2)	

Age (years)	Less than 40	619/1140 (54.3)	369/619 (59.6)	*p*=0.01^*∗*^, 3.4 (1.9–6.1)
	40 and above	521/1140 (45.7)	441/521 (84.6)	

Gender	Male	318/1140 (27.9)	224/318 (70.4)	*p*=0.20, 1.4 (0.8–2.7)
	Female	822/1140 (72.1)	586/822 (71.3)	

Comorbidity	Yes	428/1140 (37.5)	422/428 (98.6)	*p*=0.01^*∗*^, 5.2 (2.1–12.9)
	No	712/1140 (62.5)	388/712 (54.5)	

*Note.* ^*∗*^ *p* value is statistically significant. CI, confidence interval; *N*, total number; OR, odds ratio.

**Table 2:** Descriptive statistics of the overall prescription profile (*N* = 1140) at the Edna Adan University Hospital, between August 28, 2019, and August 27, 2020.

No. of drugs/prescription	Frequency	Percent
One	332	29
Two	431	37.8
Three	259	22.7
Four	82	7.2
Five	22	1.9
Six	7	0.7
Ten	7	0.7

**Table 3:** Descriptive statistics of the overall prescription profile (*N* = 1140) at the Edna Adan University Hospital from August 28, 2019, and August 27, 2020.

WHO's core indicators	Frequency of WHO's core indicator values (number (percent))				
	None	One	Two	Three	Four
Frequency of prescriptions with brand name drugs (%)	412 (36.1)	567 (49.9)	139 (12.2)	13 (1.1)	7 (0.6)
Frequency of prescriptions with antibiotics (%)	510 (44.7)	577 (50.6)	39 (3.4)	14 (1.2)	0 (0)
Frequency of prescriptions with injections (%)	913 (80.1)	200 (17.5)	20 (1.8)	7 (0.6)	0 (0)

**Table 4:** Description of the most common cases diagnosed and drugs prescribed at the Edna Adan University Hospital, between August 28, 2019, and August 27, 2020.

Diagnosis	Treatment		Number of cases	Percent
Infection	Urinary tract infection	Ciprofloxacin,	100	3.9
		Doxycycline	75	3.0
	Nonspecified infection	Amoxicillin	140	5.5
		Augmentin	231	9.1
		Ceftriaxone	259	10.2
	Wound infection	Ampiclox®	250	9.9
	Vaginal infection	Co-trimoxazole	180	7.1
	Skin infection	Gentamicin	105	4.1
	Oral candidiasis	Nystatin	61	2.4

Hypertension		Nifedipine	55	2.2
		Losartan	57	2.2
		Enalapril	60	2.4
		Propranolol	53	2.1
		Furosemide	42	1.6

Diabetes	Type I	Insulin	77	3.0
	Type II	Glibenclamide	54	2.1
		Metformin	61	2.4

GI upset	Gastritis	Omeprazole, Gaviscon	103	4.0
	Peptic ulcer disease (PUD)	Omeprazole	61	2.4
	H. Pylori-induced PUD	Triple therapy (amoxicillin, metronidazole, and omeprazole)	15	0.6
	Constipation	Lactulose	15	0.6
	Diarrhea	Loperamide	8	0.3
	Vomiting	Metoclopramide	46	1.8

Anemia		Pregnacare®, folic acid	203	8

Hormonal disorders	Heavy menstruation	Primolut-N® (norethisterone)	92	3.6
	Deficiencies	Duphaston®	61	2.4

Pain	General pain	Paracetamol	37	1.5
	Depression	Amitriptyline	15	0.6

Respiratory disorders	Asthma	Salbutamol	23	0.9

Total			2539	100

## Data Availability

The data used to support the findings of this study are available upon request.

## Supplementary Materials

Supplementary Materials Three supplementary materials were attached. They include Supplemental file 1, which contains the checklist used for data extraction from the medical records and prescriptions; Supplemental file 2, which is about the WHO prescribing indicator form; and Supplemental file 3, which contains the selected WHO core drug use indicators and their recommended standard values.
