# Supplementary material for: Assessment of Polypharmacy, Drug Use Patterns, and Associated Factors at the Edna Adan University Hospital, Hargeisa, Somaliland
Source: J Trop Med. 2022 Aug 29;2022:2858987. doi: 10.1155/2022/2858987 (PMC9444466; doi:10.1155/2022/2858987)
Supplement: Supplementary Materials — Three supplementary materials were attached. They include Supplemental file 1, which contains the checklist used for data extraction from the medical records and prescriptions; Supplemental file 2, which is about the WHO prescribing indicator form; and Supplemental file 3, which contains the selected WHO core drug use indicators and their recommended standard values. [file 2858987.f1.zip › 2858987.f1/Supplemental File 1-Check List used for data extraction from the medical records and prescriptions.docx]

# Supplement 1: Check List used for data extraction from the medical records and prescriptions

1. **Demographic characteristics of the patients** (Put the required information in the blank spaces or “X” in the boxes)

Patient ID: _______________

Age (in years):

Gender: Male Female

Admitted/visited ward: In patient

1. **Drug use information** *(Put “X” in the boxes)*

a) Total number of drugs in the patient prescription

One

Outpatient

Six

Two Seven

Three Eight

Four Nine

Five Ten

1. **The WHO Core Drug Use Indicators** *(Put “X” in the boxes)*
   1. *Presence of at least one antibiotic in the prescription. Mark X in the boxes.*

No Yes

- 1. *Presence of at least one injectable form of medicine in the prescription*

No Yes

- 1. *Presence of at least one brand name prescribed in the prescription*

: No Yes

1. *T***he WHO facility indicators (***Put “X” in the boxes)*
   1. *availability of an essential drug list (EDL); No Yes*
   2. *Standard Treatment Guideline (STG); No Yes*
   3. *National Drug Formulary; No Yes*
