# Supplementary material for: Assessment of Polypharmacy, Drug Use Patterns, and Associated Factors at the Edna Adan University Hospital, Hargeisa, Somaliland
Source: J Trop Med. 2022 Aug 29;2022:2858987. doi: 10.1155/2022/2858987 (PMC9444466; doi:10.1155/2022/2858987)
Supplement: Supplementary Materials — Three supplementary materials were attached. They include Supplemental file 1, which contains the checklist used for data extraction from the medical records and prescriptions; Supplemental file 2, which is about the WHO prescribing indicator form; and Supplemental file 3, which contains the selected WHO core drug use indicators and their recommended standard values. [file 2858987.f1.zip › 2858987.f1/Supplemental File 3-The Selected Core WHO Drug Use Indicators and their recommended standard Values.docx]

**Supplement 3: The Selected Core WHO Drug Use Indicators and their recommended standard Values (Reference 17)**

| **Core WHO Drug Use Indicator** | **Standard Values** |
| --- | --- |
| 1. Average number of drugs per encounter | 1.6 - 1.8 |
| 2.Percentage of encounters with an antibiotic prescribed | 20.0 - 26.8 |
| 3.Percentage of encounters with an injection prescribed | 13.4 - 24.1 |
| 4.Percentage of drugs prescribed by generic name | 100.0 |
| 5.Percentage of drugs prescribed from EML or formulary | 100.0 |
