# Supplementary figures and images for: Assessment of Polypharmacy, Drug Use Patterns, and Associated Factors at the Edna Adan University Hospital, Hargeisa, Somaliland
Source: J Trop Med. 2022 Aug 29;2022:2858987. doi: 10.1155/2022/2858987 (PMC9444466; doi:10.1155/2022/2858987)

**Supplement 2: WHO, Prescribing indicator form.**


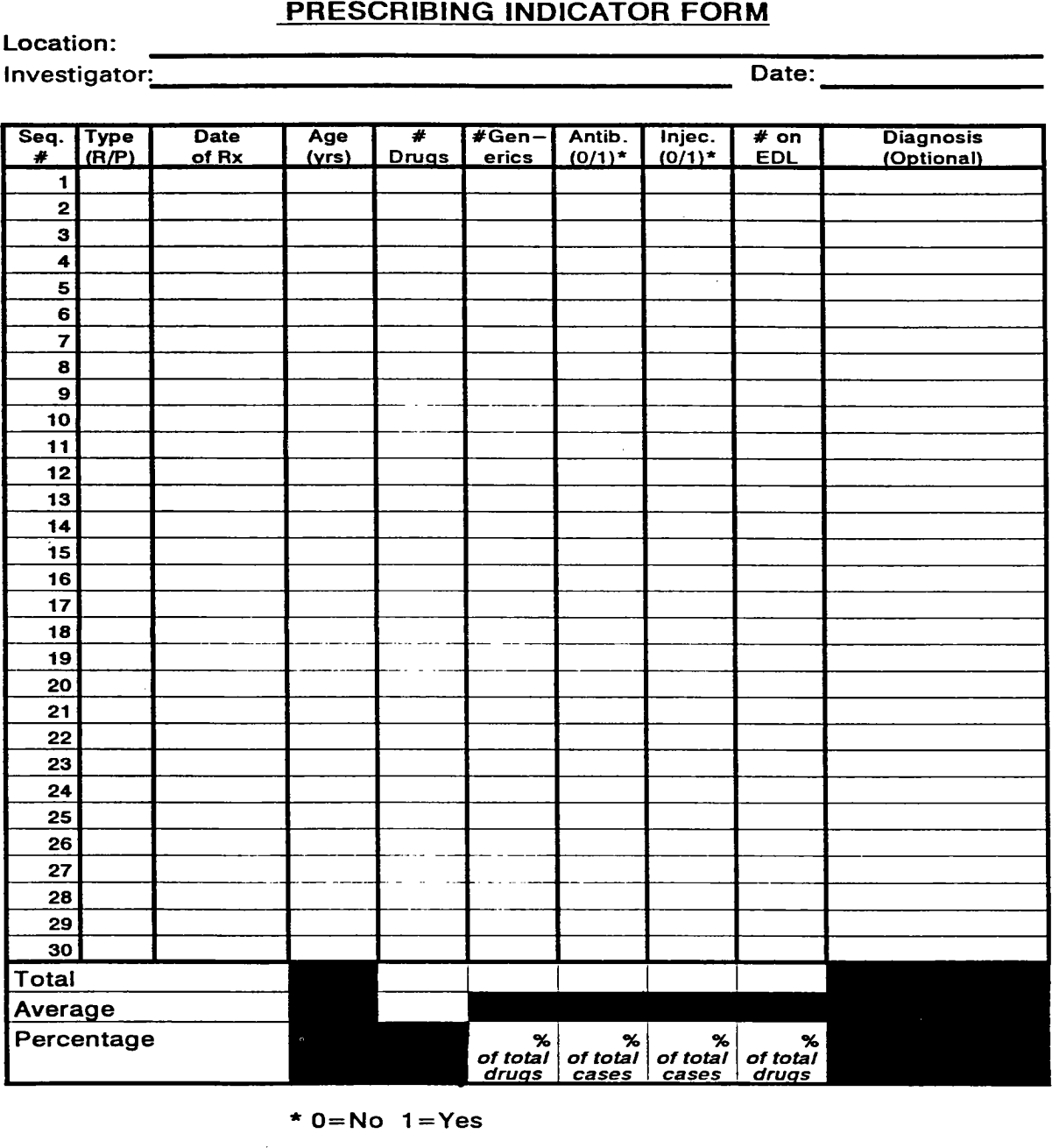

Supplement: Supplementary Materials — Three supplementary materials were attached. They include Supplemental file 1, which contains the checklist used for data extraction from the medical records and prescriptions; Supplemental file 2, which is about the WHO prescribing indicator form; and Supplemental file 3, which contains the selected WHO core drug use indicators and their recommended standard values. [file 2858987.f1.zip › 2858987.f1/Supplemental File 2-WHO, Prescribing indicator form..docx]
